# Editorial: One Health approaches to combat infectious diseases

**DOI:** 10.3389/fmicb.2023.1321134

**Published:** 2023-11-07

**Authors:** Kamran Zaman, Siraj Ahmed Khan, Soraya Chaturongakul, Biswajeet Sahoo, Ângela Novais

**Affiliations:** ^1^Indian Council of Medical Research-National Institute of Traditional Medicine Belagavi (ICMR-NITM Belagavi), Belagavi, India; ^2^Indian Council of Medical Research-Regional Medical Research Centre Dibrugarh (ICMR-RMRC Dibrugarh), Dibrugarh, India; ^3^Institute of Molecular Biosciences, Mahidol University, Nakhon Pathom, Thailand; ^4^Department of Microbiology, Vardhman Mahavir Medical College and Safdarjung Hospital, New Delhi, India; ^5^UCIBIO – Applied Molecular Biosciences Unit, Department of Biological Sciences, Faculty of Pharmacy, University of Porto, Porto, Portugal; ^6^Associate Laboratory i4HB - Institute for Health and Bioeconomy, Faculty of Pharmacy, University of Porto, Porto, Portugal

**Keywords:** One Health approaches, infectious diseases, pathogens, zoonotic disease, risk assessment, antimicrobial resistance, global health, multisectoral approach

The twenty-first century is the stage of major global transformations and challenges that ultimately affect the efficiency of health systems. Changes in human demographics, natural ecosystems and the climate created new opportunities for the emergence, re-emergence and spread of new or old infectious diseases around the globe. The COVID-19 pandemic, outbreaks of vector-borne or zoonotic diseases and antimicrobial resistance (AMR) constitute examples of current global health threats that are mutually shared by humans, animals, and the environment (One Health Basics, 2018). Although these are thoroughly recognized by different stakeholders, mechanisms for the prevention and control of infectious diseases are highly heterogeneous across institutions and geographies. Thus, effective prevention and control of these infectious diseases challenges require core competencies and concerted actions in humans, animals and the environmental sector to prevent future pandemics and to promote health sustainability (Laing et al., 2023).

The concept of “One Health” relies on recognizing the interconnection and interdependence between the health of humans, animals, plants, and their shared environment and thus has the potential to provide holistic and integrated solutions (One Health Basics, 2018; Laing et al., 2023). It encompasses a collaborative, multisectoral and transdisciplinary approach that operates at local, regional, national, and global levels to achieve optimal health outcomes. To enhance global action on the topic, the heads of the Quadripartite organizations encompassing the Food and Agriculture Organization of the United Nations (FAO), the United Nations Environment Programme (UNEP), the WHO, and the World Organization for Animal Health (WOAH) – launched in 2022 the first One Health Joint Plan of action to create an integrated framework and global capacity for infectious diseases control (including prevention, prediction, detection and response) (FAO et al., 2022). The predicted actions are expected to improve global health and health sustainability and contribute to the Sustainable Development Goals of the 2030 Agenda (https://sdgs.un.org/goals). Public awareness and engagement are guaranteed by global initiatives such as the international “One Health Day” held on the 3rd November.

The Research Topic on “*One Health approaches to combat infectious diseases*” at *Frontiers in Microbiology* aims to disseminate scholarly research and review papers of exceptional quality on key topics related to One Health initiatives to tackle infectious diseases. Furthermore, it aims to bring attention to recent discoveries in the field and provide a lens on potential opportunities for future advancements. The manuscripts included in this Research Topic are expected to stimulate discussion within the infectious diseases community, leading to the implementation of evidence-based practices in clinical, public health, and policy domains. It comprises four articles, consisting of one review, one mini-review, one opinion article and one original research article.

Marutescu et al. provided a comprehensive review of livestock farming and manure application practices in the emergence, accumulation and spread of antibiotic-resistance genes (ARG) and antibiotic-resistant bacteria (ARB) in the environment, still the least explored sector. The authors noted that composting seems to be the most effective practice that can reduce over 80% of antibiotic resistance genes, compared to sludge anaerobic digestion or lagoon storage, the efficiency of which depends on land microbiome composition which must be studied before and after manure application. The authors recognized knowledge gaps associated with the lack of data from commercial farms and difficulties in comparison between studies. They claim that subsequent studies should evaluate the impact of manure treatment methods on AMR in natural ecosystems and the food chain using harmonized methods across several geographical locations to achieve reliable and comparable outcomes. To clarify the fate of antibiotic residues, ARG and ARB during different types of manure treatment are open questions that need to be addressed by comprehensive risk assessment studies to safeguard exposure to vegetables, the food chain and humans.

Kapoor et al. provided a mini-review concerning One Health aspects related to milk-borne diseases and suggested that veterinary, legal, administrative, and health solutions are needed to reduce milk-borne diseases. While milk-borne diseases account for 4% of global foodborne infections affecting people of all ages and occupations, it has been a neglected route of infection for zoonotic diseases. The authors elaborated on the risk of exposure to humans posed by commercial milk pooling and processing practices and subsequent quality control failures that can facilitate the introduction of infectious organisms during processing and distribution. Education of dairy producers, milk handlers, and consumers about milk-borne diseases, their risks, and effective prevention is suggested as a required measure. Furthermore, the authors bring the attention of policy-makers to putative cost-effective One Health strategies to reduce milk-borne infections such as animal immunization, pasteurization, quality control in commercial milk processing, and consumer hygiene.

Smith et al. performed a longitudinal study that looked at how antimicrobial usage (AMU) affects AMR occurrence and persistence in animal farms over time. In this study, 14 cattle, sheep and pig farms in the South of England were sampled three times over a year to get information on AMR Enterobacterales in the feces of those animals using whole genome sequencing, AMU, and husbandry or management practices. The authors found that AMR, and specifically multidrug-resistant isolates, was more frequently detected in pig farms than in farms with other livestock animals and this was not always associated with high AMU usage. Sheep farms had a very low AMU and yielded the lowest incidence of AMR phenotypes and genes. On the other hand, AMR bacteria were always lower on cow farms than on pig farms, even on farms with relatively high AMU. The data presented highlight the complexity and combinatorial nature of factors other than AMU that play a role in the persistence of AMR bacteria on farms.

Beato and Veneroso analyzed the animal health industry's Nagoya Protocol (NP) implementation issues. As an extension of the Convention on Biological Diversity (CBD), the Nagoya Protocol (NP) took effect on October 12, 2014. The protocol regulates access to genetic resources (GRs) and the sharing of their benefits internationally. People must traverse many domestic access and benefit-sharing (ABS) measures. Thus, they must carefully assess each business to establish if it falls within these national standards. The authors have shown that international and national animal health organizations and institutions must act quickly to build a new access and benefit-sharing (ABS) mechanism for animal genetic resources. The authors recommend adding animal microbes to this method to replace the bilateral non-proliferation system. Standardized global benefit-sharing policies based on ABS multilateral systems from other international venues are offered.

The research studies on this Research Topic emphasized the importance of the One Health approach to combat infectious diseases in sectors or specific sources of disease that have been overlooked so far. One-Health-based infectious diseases management has shown promise in controlling food-related outbreaks and antibiotic resistance rates in humans and animals [EFSA (European Food Safety Authority) and ECDC (European Centre for Disease Prevention and Control), 2023] but increased and concerted efforts from the veterinary field and a higher involvement of the environmental sector are needed. Furthermore, it is clear the importance and urgency of comprehensive risk assessment studies based on harmonized methodologies across representative sectors and geographies to support the design and implementation of efficient One Health-based policies.

## Author contributions

KZ: Conceptualization, Writing—original draft, Writing—review & editing. SK: Writing—review & editing. SC: Writing—review & editing. BS: Writing—review & editing. ÂN: Conceptualization, Writing—original draft, Writing—review & editing.
